# Weakened pacific overturning circulation, winter monsoon dominance and tectonism re-organized Japan Sea paleoceanography during the Late Miocene global cooling

**DOI:** 10.1038/s41598-022-15441-x

**Published:** 2022-07-20

**Authors:** Kenji M. Matsuzaki, Masayuki Ikeda, Ryuji Tada

**Affiliations:** 1 Atmosphere and Ocean Research Institute, The University of Tokyo, 5-1-5 Kashiwanoha, Kashiwa, Chiba 277-8564 Japan; 2 Department of Earth and Planetary Science, Graduate School of Science, The University of Tokyo, 7-3-1, Hongo, Bunkyo-ku, Tokyo, 113-0033 Japan; 3 Institute for Geo-Cosmology, Chiba Institute of Technology, 2-17-1 Tsudanuma, Narashino, Chiba 275-0016 Japan

**Keywords:** Climate sciences, Palaeoceanography

## Abstract

The Late Miocene global cooling (LMGC; approximately 7.9–5.8 Ma) was associated with remarkable changes in monsoon dynamics, biogenic bloom in the global oceans, and the rise of modern ecosystems at the expense of old biota. However, the possible linkage between the environmental changes and ecosystem shifts during the LMGC is still debated. In this paper, we show the high-resolution changes in the fluxes of selected radiolarian species, suggesting a drastic reorganization in the paleoceanography and ecosystem in the Japan Sea during the LMGC. The endemic radiolarian *Cycladophora nakasekoi* dominated the Japan Sea until 7.4 Ma when the Japan Sea sediment changed from dark radiolarian-rich sediment to organic-poor diatom ooze. Changes in the fluxes of *C. nakasekoi* and *Tricolocapsa papillosa*, the latter related to changes in the Pacific central water (PCW), show 100, 200, and ~ 500 ka cycles with their high flux mostly within the darker sediment intervals during the low-eccentricity period until 7.4 Ma, suggesting that orbitally paced PCW inflow might have been the major nutrient source into the Japan Sea. At about 7.4 Ma, these species decreased at the expense of increased *Larcopyle weddellium*, a radiolarian related to the North Pacific intermediate water (NPIW), and *Cycladophora sphaeris*, a subarctic radiolarian species, implying a decrease in PCW inflow and an increase in the inflow of NPIW and subarctic shallow water. Such a change would have been related to the LMGC-induced weakening in the Pacific Meridional overturning circulation and the southward shift of the subarctic front due to intensified East Asian winter monsoon. Such a drastic reorganization in the hydrography in the Japan Sea probably caused changes in nutrient provenance from the PCW to the NPIW and resulted in faunal turnover, marked by the disappearance of the old regional and endemic faunal components, such as *C. nakasekoi*.

## Introduction

The late Miocene global cooling (LMGC) between 7.8 and 5.8 Ma was a climatic event associated with ice sheet formation in Greenland, intensified winter monsoon, and biogenic bloom in the global oceans^1–3^. Additionally, major turnover in marine biota, such as phytoplankton and marine mammals, occurred during the late Miocene and led to the rise of the modern ecosystem^4,5^. However, the timing and nature of such ecological shifts and their relationships with paleoceanographic changes are still debated partly because of the lack of high-resolution records.

In the Japan Sea, a marginal sea of the Northwest Pacific, a dominant endemic radiolarian, namely, *Cycladophora nakasekoi* became extinct, whereas the abundance of diatom increased during the LMGC^6,7^. As the dominance of an endemic species in a marginal sea reflects a particular ecological niche^8^, such a particular ecological niche probably prevailed in the Japan Sea before the LMGC. During the LMGC, the abundance of the giant shark *Carcharocles megalodon* decreased and the *Desmostylia* group, a group of semiaquatic marine mammals, became extinct in the Japan Sea^9,10^. Hence, it is likely that the zooplankton, phytoplankton, and macrofauna of the Japan Sea were affected by the LMGC.

**Figure 1:**
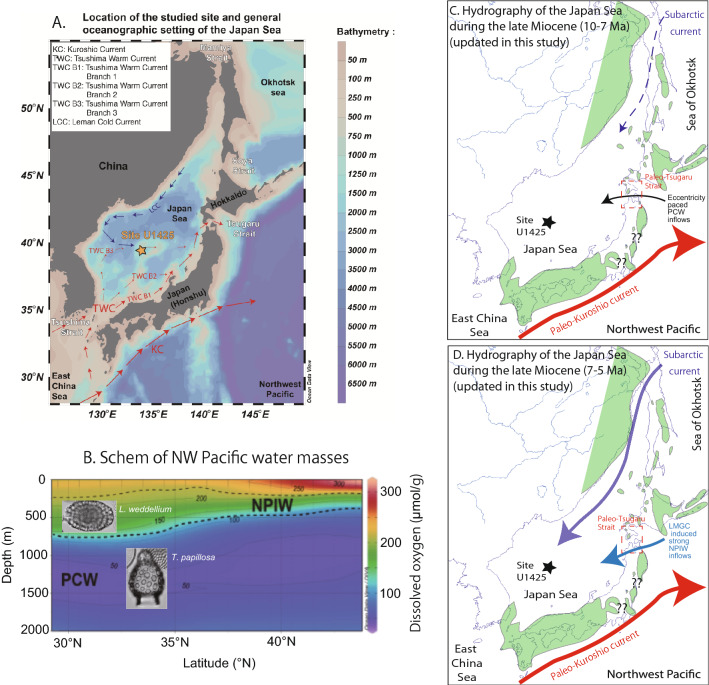
In the (**A**) panel, location of the Integrated Ocean Drilling Program (IODP) Expedition 346 site U1425. On the (**B**) panel, the modern oxygen distribution in the Northwest Pacific from latitude 30° N to 50° N based on the World Atlas Ocean (2018) and schematized inhabited water depths of *Tricolocapsa papillosa* and *Larcopyle weddellium*^39^^,^^44^. On the (**C**,**D**) panel respectively the paleoceanography of the Japan Sea reconstructed in this study between 9 and 7 Ma and 7 and 5 Ma^6^. The map in A panel and the profile in B panel was generated using Ocean Data View 4^62^ Version 5.1.0 available at https://odv.awi.de/software/download/.

This biotic turnover was even more spectacular because it is associated with the change in sedimentary facies from radiolarian-rich dark sediment to organic-poor diatom ooze; this change suggests an increased bottom water oxygenation level in the Japan Sea during the LMGC^11–13^. Today, the bottom water of the Japan Sea has the highest deep water dissolved oxygen concentration in the Pacific because it is well ventilated through winter cooling, sea-ice formation, brine rejection, subduction, and convection^14^. However, the Japan Sea paleoceanography during the late Miocene was different because the Tsugaru Strait was much deeper and wider in the late Miocene than during modern times^16,17^ (Fig. 1). The existence of Pacific-type deep water radiolarians and Nd isotopes of fish debris in the Japan Sea suggested that the deep water of the Japan Sea was connected to the North Pacific until 4.5 Ma^18,19^. In the modern North Pacific, deep waters consist of the Pacific central water (PCW) and North Pacific intermediate water (NPIW)^20^. The PCW is oxygen-poor water advected from the Southern Ocean, whereas the NPIW is relatively oxygen-rich because it forms in the Sea of Okhotsk and spreads to the low-latitude North Pacific^20,21^. Recent studies suggested that the initiation and/or intensification of the NPIW could have promoted the oxygenation of the deep water in the Japan Sea since ~ 7.4 Ma^22^.

Besides the seaway, the tectonics movements surrounding the Japan Sea could have affected the paleoceanography of the Japan Sea^12^. The sill depth of the paleo-Tsugaru Seaway shoaled from the middle bathyal water depths (1000–2000 m) to the upper bathyal water depths (500–1000 m) from 10 to 6 Ma^15^. This shoaling event is important as it could have disturbed deep water exchanges between the Japan Sea and North Pacific during the LMGC^12^. Together with the LMGC itself and its associated oceanographic changes, such tectonically driven changes in deep water exchanges could have affected the ecosystems in the Japan Sea.

In this study, we used a high-resolution species-level radiolarian accumulation rate during the interval from 9 to 5 Ma to reconstruct the regional paleoceanography because radiolarians can be used as a tracer for deep water provenance based on their (paleo)biogeographic distribution in the Northwest Pacific e.g.,^23^. Based on the biocyclostratigraphic age model of the Japan Sea sediment^24^, we examined orbital-scale changes in species-level radiolarian abundance and their relationship with paleoceanographic dynamics. We propose that the shift of the nutrient delivery system from the PCW inflow to the NPIW water masses occurred because of shoaling of the Northeast Japan Seaway, weakened Pacific Meridional overturning circulation, and intensified East Asian Winter Monsoon (EAWM) which may have caused a drastic change in the Japan Sea paleoceanography and ecosystem.

## Geographic and paleogeographic configurations of the Japan Sea

Today, the Japan Sea is a semi enclosed marginal sea located in the middle latitudes of the Northwest Pacific; its area exceeds 1,000,000 km^2^, and its mean depth is 1667 m^25^. The Japan Sea is connected to other seas with narrow and shallow straits (< 130 m in sill depth) and thus is isolated from the intermediate-to-deep water of the North Pacific. At present, the Tsushima Warm Current is the only current flowing into the Japan Sea, and its properties control the oceanographic conditions of the sea (Fig. 1)^25^. In the northwestern part of the Japan Sea, the surface water is cooled by the winter monsoon, and hence, the Japan Sea proper water, a local deep water characterized by high dissolved oxygen content and very low temperature, is formed with a residence time of approximately 100 years^25^.

The oceanographic conditions of the Japan Sea have changed throughout its tectonic history^12^. The Japan Sea is a back-arc basin that was opened by continental rifting during the late Oligocene to middle Miocene Epoch, approximately 28–13 Ma e.g.,^26^. From the occurrence of marine molluscan fossils and sedimentary facies of Neogene Japanese outcrops, it is inferred that at ~ 10 Ma (tropical–subtropical biozone N15), the Japan Sea probably was connected to the North Pacific through three seaways. One seaway was in central Japan in the region called Fossa Magna, and another one was located around the modern Mamiya Strait where mainly marine sandstone was deposited (neritic water depth, ~ 200 m) (Fig. 1)^15,16,27,28^. The third seaway was located around the modern Tsugaru Strait, where there was likely a large seaway from the central part of Hokkaido Island to the northern Honshu Island with water depths deeper than 1000 m, as indicated by the middle bathyal bio and sedimentary facies^15^. In the present paper, we refer to this strait as the paleo-Tsugaru Seaway (Fig. 1).

The outcrops from the northern part of Honshu and the southern part of Hokkaido Islands corresponding to ~ 6.5 Ma (upper N17 zone of the tropical–subtropical biozone) are characterized by upper bathyal bio-sedimentary facies, which correspond to a water depth of 500–1000 m^15^. Alternatively, numerous studies inferred that active tectonism of East Japan was caused by the subduction of Pacific plates and movement of the Izu–Bonin Arc since 10 Ma^26,29,30^. Therefore, several studies associated the shoaling of the paleo-Tsugaru Seaway to a progressive uplift due to tectonism, and this uplift gradually isolated the Japan Sea from the North Pacific e.g.,^6,12^.

## Oceanographic settings

In the modern oceanography of the North Pacific, water depths between 1000 and 2000 m in the tropics correspond to the nutrient-rich PCW (~ 1000–3000 m), which is a mixture of the water masses that originated from the Antarctic Bottom Water, Circumpolar Deep Water, and North Atlantic Deep Water e.g.,^20^. Water depths between 400 and 700 m correspond to the NPIW, formed in the Sea of Okhotsk, and spread to the low latitude in North Pacific^21^. The NPIW is also nutrient-rich but has a much lower δ^13^C value and higher concentration in dissolved oxygen than PCW (Fig. 1), and the NPIW is found at water depths shallower than the PCW^20^.

Modeling studies and Nd isotopes suggested that a modern-like thermohaline circulation was probably established in the Pacific Ocean at ~ 14 Ma^31,32^. From the increasing gradient of benthic foraminifera carbon isotope records between intermediate and deep-water masses since 13.9 Ma between the South and North Pacific, it is conceived that the PCW spread to the North Pacific in association with the expansion of the Antarctic Ice Sheet. This expansion caused a strong meridional overturning circulation (MOC) in the Pacific Ocean^32^. Additionally, the Pacific Ocean MOC was more sustained during low-eccentricity intervals because of lower sea surface temperature (SST) caused by expanded East Antarctic Ice Sheet^32^; furthermore, an expanded sea-ice around the Antarctic Ice Sheet possibly enhanced the variability in intermediate and deep-water production in the Southern Ocean, exerting a major control on the strength of the Pacific MOC^32^. In the low-latitude North Pacific, carbon and Nd isotope records and Mn/Ca records suggested that the NPIW probably existed and influenced the middle- to high-latitude regions of the North Pacific since 13.9 Ma^32^.

## Lithostratigraphy and chronology

In this study, we analyzed sediment core samples collected from site U1425 drilled during the Integrated Ocean Drilling Program (IODP) Expedition 346. Site U1425 is in the central part of the Japan Sea in the middle of the Yamato Bank (water depth: 1909 m) at 39° 29.44′ N and 134° 26.55′ E ^33^. We use the core composite depth below the seafloor (CCSF-D) Patched-Ver. 2^34^ for all sediment cores retrieved during Expedition 346. The age model is based on the cyclostratigraphy of the gamma-ray attenuation (GRA) records tuned to the short eccentricity cycle (100 ka) assuming no phase lags, in conjunction with the biostratigraphy^24,33,35^.

For this study, we briefly summarized the key features of the lithologic subunits IIIA and IIB defined at Site U1425 because they encompass the time between 9.2 and 4.1 Ma, which corresponds to the depth interval between 356 and 137 m (CCSF-D). Lithological subunit IIIA corresponds to the depth interval between approximately 356 and 262 m (CCSF-D) (i.e., approximately 9.2 to 7.36 Ma)^24,34^. This subunit is rich in terrigenous material and is characterized by decimeter- to meter-scale cyclic alternations of dark gray diatom ooze and moderately bioturbated clayey diatom ooze. Frequent laminations are observed within subunits IIIA, up to 287 m CCSF-D, which corresponds to 8.05 Ma^24^. At 287 m CCSF-D (8.05 Ma) and 321 m CCSF-D (8.75 Ma), a thinly laminated interval span for more than 1 m^24^. Conversely, subunit IIB at approximately 262–137 m CCSF-D, which corresponds to the time interval between 7.35 and 4.1 Ma, is dominated by heavily bioturbated brownish diatom ooze.

## Analysis of radiolarians

In this study, we proposed to estimate total radiolarian and selected species accumulation rates (skel. cm^−2^. ka^−1^) (*Cycladophora nakasekoi, Cycladophora sphaeris, Larcopyle weddellium* and *Tricolocapsa papillosa*), to reconstruct the paleoceanography of the Japan Sea during the Late Miocene. Species accumulation rates (skel. cm^−2^. ky^−1^) has the advantage to be more quantitative than relative abundances and because radiolarians are floating organisms transported by water masses we assumed it can better monitor specific water mass changes than relative abundances. To estimate accumulation rates of total radiolarian and selected species we need to know the absolute abundance of total radiolarian (skel. g^−1^) and species relative abundances (%).

The radiolarian absolute abundances (skel. g^−1^) and *C. nakasekoi* (%) have only been estimated for 66 samples between 9.0 and 5.3 Ma^6^, while the relative abundances (%) of *Cycladophora sphaeris, Larcopyle weddellium* and *Tricolocapsa papillosa* were estimated for 157 samples^36^ to reconstruct the Sea Surface Temperature (SST) of the Japan Sea between 9.0 and 5.3 Ma. When estimating SSTs, slides for quantitative studies (Q-slide) allowing the estimates of absolute abundances in 1 g of dry sediment were not mounted because in general Q-slides do not have enough specimens to conduct proper assemblages’ analysis in the Japan Sea (< 300 specimens)^6,37^. Thus, in this study the 91 sediment samples collected from Site U1425 used for SST estimates^6^ were proceed again for mounting radiolarian Q-slides and estimate total radiolarian absolute abundances (skel.g^−1^) following the protocol established for IODP Expedition 346^37^. In addition, we also estimate *C*. *nakasekoi* relative abundances (%) for these 91 samples. Thus, the estimated radiolarian absolute abundances and *C. nakasekoi* relative abundances (%) are original to this study. The protocol is as follows:

For the 91 samples, we estimated the absolute abundances of total radiolarians and those of *C*. *nakasekoi* in terms of the number of skeletons per gram of dry sediment collected at IODP Site U1425 between 179 and 331 m CCSF-D. We followed the methodology established for the Japan Sea sediment^37^. Briefly, the samples were freeze-dried and then treated with diluted hydrogen peroxide (10%) and hydrochloric acid (5%) to remove organic and calcareous matter. The undissolved residue in each sample was sieved through a 45 µm screen. Once the undissolved residue was washed, we mounted the residue on Q-slides for quantitative radiolarian studies. To prepare Q-slides, the undissolved residue was transferred to a 100-mL beaker containing 100 mL of water. The solution was then mixed, and a 0.2 mL sample was taken from the suspension using a micropipette and dropped onto a cover glass of area 22 × 18 mm. Then, we counted all the radiolarians in a Q-slide under an optical microscope at magnifications of 100 × to 400 × . Then, the total radiolarian absolute abundances in 1 g of dry sediment were estimated using the following equation:

$${\text{AA}}_{{({\text{skel}}.{\text{ g}} - {1})}} \, = \,{1}00{\text{a}}/0.{2} {\text{g}},$$

where AA is the estimated radiolarian absolute abundances (skel. g-1), a is the number of radiolarian skeletons counted in one Q-Slide; g is the weight of the freeze-dried sample; 100 mL is the volume of water in the beaker; 0.2 mL is the volume taken using the micropipette^6^. After calculating AA, we estimated the total radiolarian accumulation rates (RAs) as follows:

$${\text{RA}}_{{({\text{skel}}.{\text{cm}} - {2}.{\text{ ky}} - {1})}} \, = \,{\text{AA}} \times {\text{R}} \times {\text{D}},$$

where the linear sedimentation rate (R, cm. ky^−1^) is estimated using cyclostratigraphy tie points^24^ and the GRA bulk density of the sediment (D, g. cm^−3^) is obtained from shipboard data^33^. Errors exist in the GRA bulk density data because of the presence of air between a core and a core liner; however, generally, the GRA bulk density tends to reflect the characteristics of each lithologic unit^33^, and it provide high-resolution records that are a better fit for estimating radiolarian fluxes rather than the low-resolution dry bulk density data.

Then, we estimated the accumulation rates of each species. The remaining residue, which was not taken up with the micropipette, was mounted onto a cover glass of size 22 × 40 mm applying the decantation method i.e.,^37,38^ and relative abundances (%) of *C. nakasekoi* were estimated inside a population containing 300 specimens at least. Then, we estimated the *C*. *nakasekoi* accumulation rates as follows:

$$Cycladophora \, nakasekoi\,{\text{AR}}\, = \,{\text{RA}} \times C.\,nakaseko{\text{i }}\left( \% \right).$$

To monitor the provenance of the water masses in the Japan Sea during the late Miocene, we also estimated the accumulation rates of selected subarctic shallow species and intermediate water species following the nomenclature established for the Northwest Pacific^6,23,39^. The subarctic shallow species only comprised *Cycladophora sphaeris*, and the intermediate water species group comprised *Larcopyle weddellium* and *Tricolocapsa papillosa*. All the selected radiolarian species from site U1425 were illustrated, and their faunal references were provided in previous studies^40^. However, the species name of *Tricolocapsa papillosa* (previously *Carpocanarium papillosum*) was amended following the latest nomenclature^64^. The relative abundances of *C*. *sphaeris*, *L*. *weddellium*, and *T*. *papillosa* are derived from a previous study^36^ and was estimated inside a population containing 300 specimens at least as well. We estimated their accumulation rates as follows:

$$Cycladophora \, sphaeris\,{\text{AR}}\, = \,{\text{RA}} \times C. \, sphaeris\left( \% \right),$$

$${{Larcopyle\,weddellium\,AR}}\, = \,{\text{RA}}\, \times \,{{L}}.\,{{ weddellium }}\left( \% \right),$$

$${{Tricolocapsa\,papillosa\,AR}}\, = \,{\text{RA}}\, \times \,{{T}}.\,{{ papillosa }}\left( \% \right).$$

Hence, we obtained a new dataset of total radiolarian and selected species accumulation rates of 157 samples. Our sampling resolution is approximately 16 ky for the time interval between 9.0 and 7.0 Ma and 30 ka between 7.0 and 5.3 Ma.

## Spectral analysis

To examine the orbital-scale changes in radiolarian abundance and other paleoceanographic proxies, we performed a wavelet analysis using a modified series of Matlab algorithms^41^. This program can identify whether the peaks in a spectrum of a time series are significant against the red-noise (autoregressive lag1) background spectrum. For the same site, we also conducted spectral analyses on sediment reflectance data (L*)^33^, which is a semiquantitative proxy for the total organic carbon (TOC)^40^, and sea-level change^43^ for comparison with the radiolarian records.

## Results

**Figure 2:**
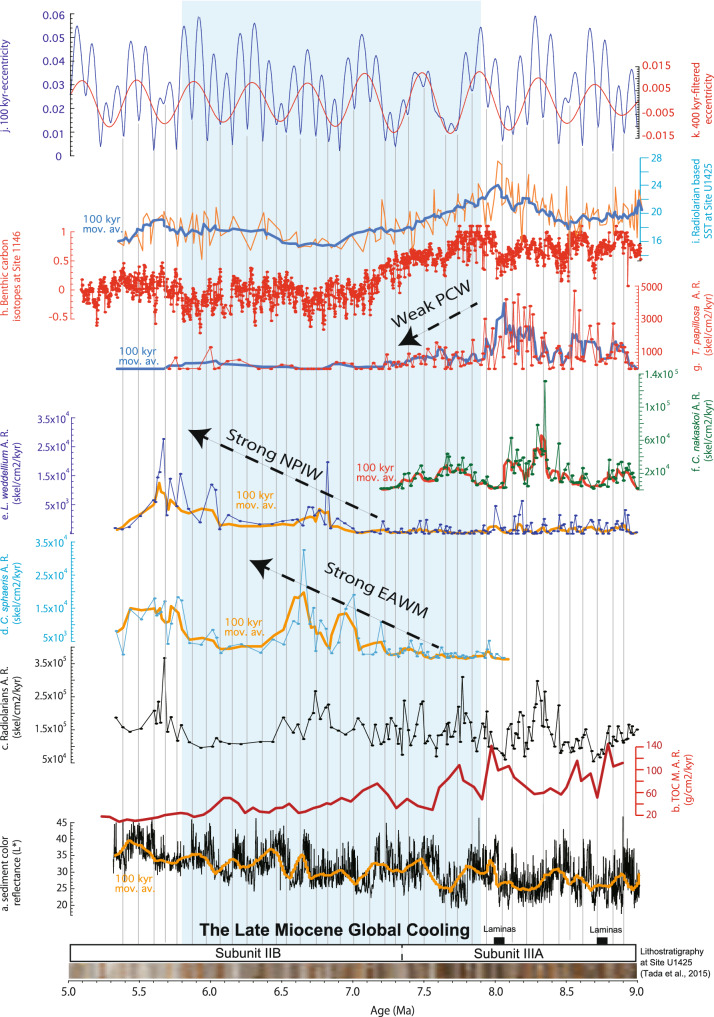
Paleoceanographic evolution of the Japan Sea across the late Miocene global cooling (LMGC). In the (X) axis, the age memory with lithological subunits at site U1425 and their lithologies^33^: (**a**) Sediment color lightness (L*) at site U1425^33^ and (**b**) mass accumulation rates (g. cm^−2^. ky^−1^) of the total organic carbon (TOC) accumulated on the seafloor at IODP site U1425^22^ with age updated model^24^. Fluxes (accumulation rates in units of skel^−1^.cm^−2^. ky^−1^) of (**c**) the total radiolarians (black), (**d**) *Cycladophora sphaeris* (light blue), (**e**) *L. weddellium* (dark blue), (**f**) *Cycladophora nakasekoi* (green), and (**g**) *T. papillosa* (red), all in this study. (**h**) Benthic carbon isotope stratigraphy at ODP 1146, South China Sea^3^, (**i**) radiolarian-based winter-time sea surface temperature in the Japan Sea at site U1425^36^, (**j**) 100 ky eccentricity cycle (63), and (**k**) 400 ky filtered eccentricity cycle^63^. Hairlines indicate projections of the eccentricity minima. The graphs were generated using KaleidaGraph software version 4.5 (https://www.synergy.com).

For the studied time interval (9.0–5.3 Ma), the total RAs have a mean value of 9.4 × 10^4^ skel. cm^−2.^ ky^−1^ (N = 157, Stand. dev. = 5.0 × 10^4^; Std. error = 4 × 10^3^) (Fig. 2c, Table 1). The maximum total RAs were recorded at 5.65 Ma with a value of 3.17 × 10^5^ skel. cm^−2^. ky^−1^ (Fig. 2c, Table 1). *C. nakasekoi*, which is the dominant radiolarian species between 9.0 and 7.1 Ma^6^, has a mean AR of 1.4 × 10^4^ skel. cm^−2.^ ky^−1^ (N = 157; Stand. dev. = 1.7 × 10^4^; Std. error = 1.4 × 10^3^). The AR of *C. nakasekoi* (1.3 × 10^5^ skel. cm^−2.^ ky^−1^) corresponded to 8.33 Ma (Fig. 2f, Table 1). Previously, the last occurrence (LO) of *C. nakasekoi* was estimated at 7.4 (± 0.1 Ma) Ma^6,35^; by contrast, we found *C. nakasekoi* until 7.05 (± 0.015) Ma with moderate peaks at 7.3 and 7.2 Ma (Fig. 2). *T. papillosa* has a mean AR of 828 skel. cm^−2.^ ky^−1^ (N = 157, Stand. dev. = 1105; Std. error = 88) (Fig. 2g, Table 1). The maximum AR of *T. papillosa* was 6649 skel. cm^−2.^ ky^−1^ at 8.54 Ma (Fig. 2g, Table 1). *C. sphaeris* has a mean AR of 2752 skel. cm^−2.^ ky^−1^ (N = 157, Stand. dev. = 5158; Std. error = 411), and its maximum of 3.2 × 10^4^ skel. cm^−2.^ ky^−1^ is recorded at 6.60 Ma (Fig. 2d, Table 1). Lastly, *L. weddellium* has a mean AR of 1.6 × 10^4^ skel. cm^−2.^ ky^−1^ (N = 157, Stand. dev. = 2530; Std. error = 201), with the maximum of 2.58 × 10^5^ skel. cm^−2.^ ky^−1^ at 5.63 Ma (Fig. 2e, Table 1).

**Table 1:** Basic statistics on used environmental variables in this study.

Summary statistics	RA (skel/cm^2^/kyr)	*C. nakasekoi* AR (skel/cm^2^/kyr)	*T. papillosum* AR (skel/cm^2^/kyr)	*C. sphaeris* AR (skel/cm2/kyr)	*L. weddelium* group AR (skel/cm2/kyr)	Sediment colour reflectance (L*)
N	157	157	157	157	157	3061
Min	4899.271	0	0	0	0	17.5
Max	317,028.1	129,932.9	6649.929	32,606.63	16,348.71	46.7
Sum	1.48E + 07	2,241,815	130,007.3	432,146.4	258,833.5	93,788.4
Mean	94,007.53	14,279.08	828.0719	2752.525	1648.621	30.63979
Std. error	4054.488	1402.909	88.22664	411.7313	201.9603	0.09737839
Variance	2.58E + 09	3.09E + 08	1,222,079	2.66E + 07	6,403,710	29.02609
Stand. dev	50,802.59	17,578.4	1105.477	5158.979	2530.555	5.387587
Median	85,761.32	8844.921	460.2794	0	699.4964	30.4
25 prcntil	58,630.25	0	0	0	217.166	26.4
75 prcntil	118,114	22,701.13	1090.436	2978.376	2119.912	34.4
Skewness	1.169598	2.538474	2.314391	2.639131	2.985749	0.2363111
Kurtosis	2.364752	11.78111	6.494868	8.295019	10.76836	-53.14539
Geom. mean	80,263.1	0	0	0	0	30.16506
Coeff. var	54.04098	123.106	133.5001	187.4271	153.4953	17.58363

The *C. nakasekoi* and *T. papillosa* fluxes were higher than their mean in the periods 8.9–8.75, ~ 8.6, ~ 8.55, ~ 8.45, 8.35–8.05, and 7.8–7.6 Ma, when the sediment L* is low (Fig. 2). The Pearson correlation coefficients of TOC (wt%) with *C. nakasekoi* and *T. papillosa* at Site U1425 were 0.56 and 0.53, respectively.

**Figure 3:**
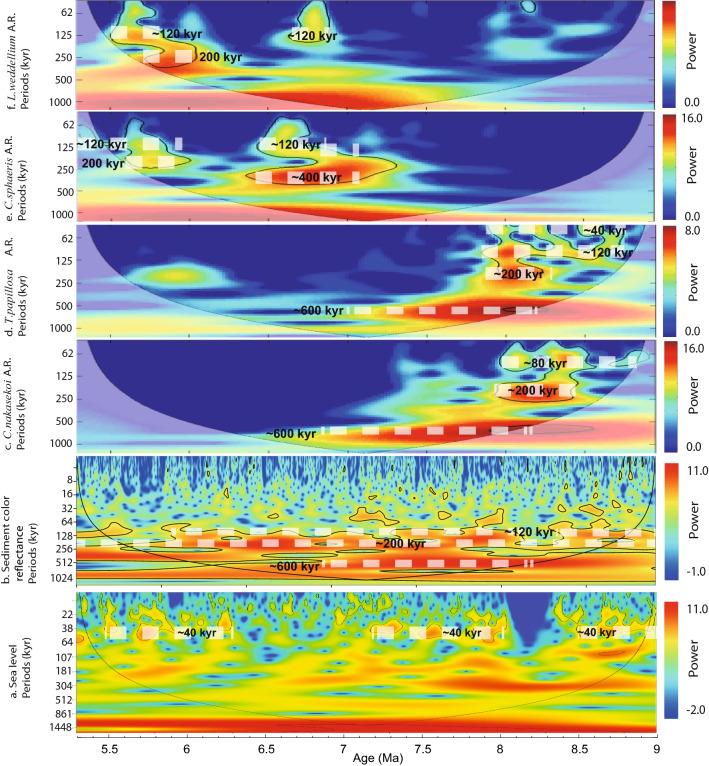
Wavelet spectra of (**a**) global mean sea-level variations^43^ and (**b**) IODP site U1425 sediment color reflectance (L*)^33^. Fluxes (accumulation rates in units of skel^−1^.cm^−2^*.*ky^−1^) of (**c**) *C. nakasekoi*, (**d**) *T. papillosa*, (**e**) *C. sphaeris*, and (**f**) *L. weddellium*, all in this study. Area with black outline indicate statistically significant frequencies (*p* < 0.05). The wavelet was generated using MATLAB Software version R2022a. (https://jp.mathworks.com) and PAST4 Software version 1.0.1 (https://www.nhm.uio.no/english/research/infrastructure/past/).

The spectral analysis of the AR of these species revealed strong 80-ky cycle between 8.8 and 8.0 Ma and 200 ka cycle between 8.5 and 8.0 Ma for *C. nakasekoi* (Fig. 3c). The 40 and 100 ky cycles were strong for *T. papillosa* between 8.8 and 7.9 Ma (Fig. 3d). For *T. papillosa*, a 200-ky cycle was also significant between 8.4 and 8.0 Ma (Fig. 3d). The AR of *L. weddellium* shows moderate to weak 100 ky cycles during 8.7–8.4, 6.9–6.6, and 5.9–5.5 Ma and ~ 200 ky cycles between 8.5 and 8.0 Ma and between 6.0 and 5.8 Ma (Fig. 3f). The AR of *C. sphaeris* also show moderate 100 ky cycles for the periods 6.9–6.4 and 5.9–5.5 Ma and 200–400 ky cycles between 7.2 and 6.3 Ma (Fig. 3e). The spectral analysis of the sediment L*^33^ also showed constant strong 200 ky and ~ 500 ky cycles between 8.7 and 6 Ma and strong 100 ky cycles in 9.0–8.4, 8.1–7.4, and 6.4–6 Ma (Fig. 3b). The 40-ky cycle is stronger at ~ 8.6, 8.0, and 7.3–7.0 Ma (Fig. 3b).

## Discussion

### Orbitally paced PCW inflows into the Japan Sea between 9.0 and 7.8 Ma

*T*. *papillosa* is regarded to be a marker of the inflow of intermediate-to-deep waters of the North Pacific into the Japan Sea that occurred between 10 and 7 Ma^6^. In recent studies on the plankton in the subtropical Northwest Pacific (Kyushu Paleo-Ridge), living *T. papillosa* specimens were observed at water depths of 1000–2000 m^39^; furthermore, in the East China Sea, few living specimens were observed at water depths of approximately 700 m, close to the seafloor^44^. In the modern oceanography of the North Pacific, the water depths of 1000–2000 m in the tropics correspond to the PCW (~ 1000 to ~ 3000 m), which is a mass of nutrient-rich and poorly oxygenated intermediate-to-deep water unlike the NPIW e.g.,^20^. Modeling studies and Nd isotope studies indicated that the PCW has influenced the North Pacific paleoceanography since ~ 14 Ma^31,32^. Thus, we consider *T. papillosa* to indicate PCW inflows into the Japan Sea between 8.9 and 7.0 Ma (Fig. 1).

Between 10 and 6 Ma, the Japan Sea was connected to the intermediate-to-deep water of the North Pacific only in the middle latitude region via the paleo-Tsugaru Seaway (~ 40°N) (Fig. 1); the sill depth reached the middle to upper bathyal water depths^12,15^. By contrast, the sill depths of the other seaways such as the Fossa Magna and Mamiya Straits were estimated as neritic water depths^15,28^. Thus, the PCW probably flowed into the Japan Sea through the paleo-Tsugaru Seaway at least between 10 and 6 Ma.

Changes in *T. papillosa* flux were mainly driven by the ~ 100 and ~ 200 ky cycles between 8.7 and 7.9 Ma (Fig. 3). The ~ 200 ky cycle is also observed in the middle to late Miocene δ^18^O records of Southeast Atlantic (ODP 1085) and the middle Eocene carbonate succession of Newfoundland^45,46^. The ~ 200 ky cycle was recently associated with eccentricity cycles, which is related to the gravitational interactions of Venus, Mars, and Jupiter^47^. Additionally, high *T. papillosa* flux intervals occurred at 100- and 400-ky-scale eccentricity minima between 9.0 and 7.9 Ma (Fig. 2). Thus, it is possible that PCW inflows into the Japan Sea were paced by the eccentricity cycles between 9.0 and 7.9 Ma.

The increasing gradient between the intermediate and deep-water carbon isotopes of the benthic foraminifera in the North Pacific since 13.9 Ma suggests that the spread of the PCW into the North Pacific because of a strong MOC started in the Pacific Ocean at this time^32^. The Pacific MOC is regarded to have been stronger during low-eccentricity intervals at this time because of a lower SST and an expanded sea-ice presence around Antarctica, which enhanced the variability in the production of intermediate and deep waters in the Southern Ocean and became a major controlling factor for the strength of the Pacific MOC^32^. The isotope gradients between the intermediate and deep waters in the Southern Ocean suggest that the deep water in the Southern Ocean was well-ventilated between 9.0 and ~ 7.3 Ma^48^. Hence, it is possible that PCW inflows into the Japan Sea were related to a strong Pacific MOC paced by an eccentricity cycle at least until 7.3 Ma.

The variations in *T. papillosa* fluxes in the Japan Sea also show 40 ky signals between 8.5 and 7.9 Ma (Fig. 3). During the late Miocene, the ~ 40 ka obliquity signal-regulated insolation at the high latitudes, which in turn controlled the volume of the Antarctic ice sheet and thus the global eustatic sea-level variation, which are up to ~ 40 m at this time (Fig. 3) e.g.,^43^. Variations in the global eustatic sea level perhaps influenced the inflow of PCW into the Japan Sea; however, the depth of the PCW is in the range of ~ 1000–3000 m in the North Pacific and the sill depths of the paleo-Tsugaru Seaway between mid-bathyal water depths for ~ 10 Ma^15^. Thus, it is probable that the influence of the variations in the global eustatic sea level is limited. Alternatively, changes in the volume of the Antarctic ice sheet could have influenced the rate of the Pacific Ocean MOC, which potentially regulated the inflows of PCW into the Japan Sea. Indeed, variations in the Antarctic ice sheet volume influenced the regional sea-ice expansion and the intermediate-to-deep water production rate in the Southern Ocean and thus the Pacific MOC^32^.

Like *T. papillosa*, the variations in *L. weddellium* fluxes also show significant 40 ky obliquity signals between 8.3 and 8.2 Ma and weaker signals of ~ 100 and ~ 200 ky cycles between 8.7 and 7.9 Ma (Fig. 3). *L. weddellium* is an extant species abundant in the subarctic Northwest Pacific surface sediment^23^ and inhabits water depths of 300–1000 m, corresponding to the NPIW^39,44^. Hence, we suggest that *L. weddellium* is related to the NPIW during the late Miocene in the North Pacific. Today, several mechanisms contribute to the formation of the NPIW. One component is the influence of the Sea of Okhotsk intermediate water formed by brine rejection in the Sea of Okhotsk during the melting of sea ice, which flows to the Northwest Pacific^21^. The second component is the mixing of waters from the cold Oyashio Current with the warm Kuroshio and the Tsugaru Warm Current in the mixed water region off Northeast Japan, thereby generating a cold less-saline intermediate water^49^. Given the probable absence of sea ice in the Sea of Okhotsk during the late Miocene because of an air temperature of around 13 °C according to floral assemblages^50^, the NPIW during the late Miocene was probably formed by thermal contrast and mixing of warm and cold waters at middle-to-high latitudes in the Northwest Pacific. Thus, the variations in *L. weddellium* fluxes implied orbital-scale changes in NPIW inflows into the Japan Sea probably through changes in the NPIW production rates, which are probably stronger during orbital phases (eccentricity–obliquity) with lower SST in the middle-to-high latitudes of the North Pacific.

The overall in-phase relation between the fluxes of *T. papillosa* and TOC between 8.8 and 8.0 Ma^22^ implies an increased nutrient-rich PCW supply during high *T. papillosa* flux intervals despite a lower resolution for TOC (Fig. 2). Considering darker sediment (L*) mean that there is a high TOC content and a brighter sediment (L*) mean there is a low TOC content^42^, the ~ 40, ~ 100, and ~ 200 ky eccentricity signals in sediment L* between 9.0 and 6.0 Ma^33^ may also support the claim that TOC increased because of the orbitally paced PCW supply to the Japan Sea (Fig. 2). Additionally, the interval with the high flux of *T. papillosa* at around 8 Ma corresponds to laminated intervals, indicating an anaerobic bottom water environment (< 0.1 ml/L of dissolved O_2_) e.g.,^51^ (Fig. 2). Thus, it is probable that the inflow of nutrient-rich PCW might have generated a periodic stratification of the intermediate and deep-water in the Japan Sea during the intervals of strong Pacific MOC.

### Reduction in PCW inflow into the Japan Sea because of local tectonism and weakened Pacific MOC due to the LMGC since ~ 8.0 Ma

A drastic decrease in the relative abundance of *T. papillosa* from ~ 6 to ~ 0% between 8.0 and 7.8 Ma suggests that the sill depth of the paleo-Tsugaru seaway shoaled at that time, although exact timing is not well-constrained, as sampling resolution was on the order of ~ 100 ky^6^. In this study, the high-resolution *T. papillosa* flux revealed a steady decrease between 8.0 and 5.2 Ma (Fig. 2). Because *T. papillosa* is abundant at water depths between ~ 1000 and 3000 m e.g.,^39^, the inflow of PCW into the Japan Sea may have been prevented by the shoaling of the paleo-Tsugaru Strait sill depth since ~ 8 Ma. Indeed, Northeast Japan was subject to tectonism between 10 and 4.5 Ma^29,30^, and the sill depths of the paleo-Tsugaru seaway probably shoaled, as indicated by the comparisons of the sedimentary facies and biofacies and the Nd isotopes in the Japan Sea and the North Pacific^15,19^. Although the sill depths of the paleo-Tsugaru Seaway were middle bathyal at ~ 10 Ma, probably the sill depth of the paleo-Tsugaru Seaway shoaled to the upper bathyal water depths at ~ 6 Ma^15^. Considering that the boundary between the upper and middle bathyal water depths is ~ 1000 m e.g.,^52^, a progressive shoaling of the sill depths of the paleo-Tsugaru Seaway may have prevented PCW inflows into the Japan Sea since ~ 8 Ma.

Additionally, in the South Atlantic Ocean (ODP sites 1088, 704, and 1090), the benthic δ^13^C records indicated the δ^13^C gradient between the intermediate water and deep-water masses decreased drastically at ~ 7.3 Ma^48^. This decrease in the δ^13^C gradient was associated with a reduction in the ventilation of the Southern Ocean deep waters at ~ 7.3 Ma because of a change in the contribution of deep-water from the North Atlantic to the Southern Ocean due to the LMGC and the possible glaciation of East Greenland, and may have reduced vertical mixing across the thermocline^48^. As reduced ventilation in the Southern Ocean would have reduced the Pacific MOC rates, thus the decrease in *T. papillosa* flux recorded in the Japan Sea might also be related to the decreasing ventilation of the Southern Ocean deep waters as well. In such a situation, the influence of PCW on the Japan Sea was probably prevented by two factors since 8 Ma. As proposed in previous studies, it is possible that the tectonic uplifting of Northeast Japan between 10 and 4.5 Ma steadily caused the shoaling of the paleo-Tsugaru Strait and since ~ 8 Ma, the shoaling was possibly enough for reducing the influence of the PCW in Japan Sea. Additionally, the decrease in the ventilation of the Southern Ocean deep water because of the LMGC at ~ 7.3 Ma weakened the Pacific MOC rates, and hence, the Japan Sea was likely much less influenced by the PCW since ~ 7.3 Ma.

The decreasing influence of PCW probably modified the bottom water properties and oxygenation level in the Japan Sea. Indeed, the lithology of the sediment collected at site U1425 changed at ~ 7.36 Ma from radiolarian-rich dark layers (subunit IIIA) to organic-poor diatom ooze (subunit IIB) at site U1425^24,33^ (Fig. 2). Subunit IIIA is occasionally laminated, whereas subunit IIB is heavily bioturbated^33^, indicating an increase in the benthic infaunal activity by the increase in the oxygen level of the bottom water, from an anaerobic (< 0.1 mL/L dissolved O_2_) to an aerobic (> 1.0 mL/L dissolved O_2_) condition e.g.,^51^. Therefore, sediment lithology at site U1425 suggested that it is possible that the deep water of the Japan Sea became oxygenated with the decreasing PCW inflow because the latter is low in dissolved oxygen. This hypothesis is supported by the analysis of Ba concentration in the sediment at the same site, that is, U1425, suggesting drastic changes in the Japan Sea bottom water redox condition at ~ 7.3 Ma^22^.

### LMGC-induced intensification of the EAWM and higher production rates of the NPIW since ~ 7.3 Ma

The fluxes of *C. sphaeris*, an extinct species likely related to subarctic shallow water^6,53^, increased from 7.3 Ma, until reaching its maximum at ~ 6.6 Ma (Fig. 2), implying a progressive increase in the influence of cold shallow water in the Japan Sea. In the Japan Sea, the winter SST decreased from 24 to 15 °C from 7.9 to 6.9 Ma as indicated by the extant radiolarian species^36^. Thus, it is probable that the increasing *C. sphaeris* fluxes since 7.9 Ma imply a southward shift of the subarctic front, which caused a cooling of the local SSTs (Fig. 2).

The high *C. sphaeris* fluxes main peaks occur at ca. 6.6 and 6.9 Ma during minima in eccentricity (Fig. 2d,j). The eccentricity cycles predominantly determine not only the Pacific MOC^32^ but also monsoon dynamics, as indicated by climate model results and geologic records^54^. Several studies also suggested a probable intensification of the EAWM since 7.0 Ma during the LMGC, as documented by an increasing aeolian dust deposition in the South China Sea from 7 to 6 Ma^55^ and increasing dry–cold vegetation pollen around the drainage area of the Pearl River since 8 Ma^56^. In the Japan Sea, high peaks of the *C. sphaeris* flux indicating cold surface waters, mostly occurred with colder winter SSTs (~ 15 °C)^36^, suggesting that the *C. sphaeris* fluxes probably increased with the southward shift of the subarctic front under the intensified EAWM. Previous diatom studies showed an increase in the abundances in the upwelling-related diatom, *Chaetoceros* resting spores, between 7.4 and 5 Ma^7^. Thus, we suggest it is more likely that since 7.4 Ma, under an intensified EAWM, intensified mixing of the shallow-to-subsurface waters occurred in the Japan Sea.

Conversely, fluxes of *L. weddellium*, which inhabited the water depths influenced by the NPIW today, are between 0 and 5000 skel. cm^−2.^ ky^−1^ until ~ 7.0 Ma and increase drastically (> 1 × 10^4^ skel. cm^−2.^ ky^−1^) around 6.9–6.6, ~ 6.0, and 5.8–5.4 Ma (Fig. 2). Thus, it is likely that the inflow of the NPIW into the Japan Sea begin to increase since 7.0 Ma and dramatically increase since 6.8 Ma (Fig. 2). In this study, the high fluxes of *C. sphaeris* in general fit with those of *L. weddellium*, with a Spearman’s rank correlation coefficient R of 0.62 in the period from 6.5–5.2 Ma. This finding implies that the NPIW inflow into the Japan Sea is probably related to the southern shift of the subarctic front, which is regulated by the EAWM. As it is probable that during the late Miocene, the NPIW formed because of the thermal contrast and mixing of warm and cold waters at middle-to-high latitudes in the Northwest Pacific, we suggest that possibly higher production rates of the NPIW occurred in the Northwest Pacific since 7 Ma during the episodes of a southward shift of the subarctic front due to a strong EAWM.

Lastly, nitrogen isotopes from site U1425 and total biogenic silica estimated based on X-ray fluorescence (XRF) and X-ray diffraction measurements at sites 794/797, also located in the Japan Sea, indicated a sustained increase in nutrient availability in the Japan Sea since ~ 7.4 Ma^12,22^. Today, the NPIW influences waters at 300–1000 m depths, and thus although speculative, we suggest that possibly strong EAWM northeasterly winds caused a mixing of the upper part of the NPIW with the shallow water, thus contributing to the enhanced primary productivity recorded in the Japan Sea since ~ 7.4 Ma.

### Possible impact of LMGC on ecosystems of the Japan Sea

During the late Miocene, *C. nakasekoi*, which is an endemic radiolarian, was dominant in the Japan Sea until its extinction at 7 Ma, near the coldest period in the LMGC (Fig. 2). Throughout the LMGC, the nutrient delivery system in the Japan Sea probably shifted from a PCW-derived nutrient-rich water mass, originating from the Southern Ocean to NPIW-derived well-ventilated condition because of shoaling of the paleo-Tsugaru Strait, weakening of the Pacific MOC, and intensification of the EAWM.

Previous studies indicated that relative abundances of *C. nakasekoi* were ~ 40% at site U1425 and Deep-Sea Drilling Program site 302, whose water depths were 1900 and 2400 m, respectively^6,18^; by contrast, its relative abundance was ~ 20% of the total assemblages at IODP site U1430, with a water depth of 1080 m. A backstripping of the stratigraphic sequences conducted at ODP sites 794–797 showed that the bathymetry of the major basins of the Japan Sea, such as the Yamato Basin, were close to those of today since ~ 16 Ma^57^. Hence, it is suggested that *C. nakasekoi* inhabited a water depth below the sill depth of the paleo-Tsugaru Strait (< 1000 m) in the Japan Sea, which probably isolated *C. nakasekoi* geographically from other deep-sea basins^6^. The overall similar trends and cycles of the *C. nakasekoi* and *T. papillosa* fluxes suggest that the *C. nakasekoi* flux was related to the inflow of nutrient-enriched PCW into the Japan Sea paced with Pacific MOC.

Although no direct evidence is available, it is possible that during periods of strong Pacific MOC, the PCW is thicker, and thus, it may influence water depths shallower than 1000 m, which is the upper threshold of the PCW depths today^20^. In such a situation, PCW possibly influenced the intermediate water depths of the Japan Sea during intervals of the strong Pacific MOC. Additionally, until ~ 7.0 Ma, the East Asian Summer Monsoon (EASM) climate dominated, as suggested by the Tibetan Plateau clay mineral records and XRF scan analysis from sediments collected at ODP 1146 (South China Sea)^58,59^. Today, in an area that is influenced by the summer monsoon, such as the East China Sea, productivity is controlled by the upwelling of the NPIW on the slope of the continental shelf e.g.,^60^. It is suggested that advection of the freshwater discharged by rivers in an estuarine circulation allowed NPIW to upwell to the shelf e.g.,^60^; radiolarians possibly were sensitive to this phenomenon in the last 400,000 years^61^. Thus, we speculate that during strong Pacific MOC, there is a thicker PCW layer, which possibly influenced the intermediate water of the Japan Sea. Then, because of a strong EASM, freshwater discharged by rivers surrounding the Japan Sea might have allowed a weak upwelling of the upper PCW flowing to the Japan Sea and favored the *C. nakasekoi* bloom.

Like *C. papillosa*, *C. nakasekoi* flux gradually decreased from 7.6 Ma, implying a decreased PCW inflow into the Japan Sea by the shoaling of the paleo-Tsugaru Strait and weakening of the Pacific MOC (Fig. 2). Note that the LO of *C. nakasekoi* at 7.05 Ma corresponds to the moderate peaks of the fluxes of *L. weddellium* and *C. sphaeris* and a cold winter SST below 18 °C, implying a southward shift of the subarctic front and enhanced production rates of the NPIW, intensification of the EAWM, and possibly vertical mixing of the upper intermediate-to-shallow waters. We suggest that these factors were critical for the extinction of *C. nakasekoi*.

## Conclusion

In this study, we reconstructed the high-resolution radiolarian fluxes as the unique proxy for changes in the surface-to-intermediate water in the North Pacific and the Japan Sea and discussed the hydrographic changes in the Japan Sea between 9.0 and 5.2 Ma. We focused on exchanges of intermediate waters with the North Pacific during the LMGC. Between 9.0 and 7.4 Ma, the dominance of an endemic radiolarian *C. nakasekoi* and the presence of PCW-related *T. papillosa* suggest that the Japan Sea possibly was influenced by inflows from PCW. Additionally, the Pacific MOC might have thickened the PCW during intervals of low eccentricity. In this situation, the PCW possibly influenced the intermediate water of the Japan Sea and caused episodic strong stratification of the water the Japan Sea water column allowing a good preservation of the laminated sediments in the Japan Sea during the Late Miocene. Across the LMGC, the *C. nakasekoi* and *T*. *papillosa* populations decreased, whereas numbers of NPIW species *L. weddellium* and subarctic species *C. sphaeris* increased. This faunal turnover suggests that the decreased PCW and increased NPIW influence into the Japan Sea might have been related to a weakened Pacific MOC and southward shift of the subarctic front because of the LMGC. Additionally, local tectonism characterized by the uplift of the paleo-Tsugaru Seaway probably contributed in the decreasing influence of the PCW as well.

## Supplementary Information

Supplementary Information.

## Data Availability

All data analyzed during this study are included in this published article and its supplementary information file.
